# Monumental olive trees of Cyprus contributed to the establishment of the contemporary olive germplasm

**DOI:** 10.1371/journal.pone.0187697

**Published:** 2017-11-07

**Authors:** Katerina Anestiadou, Nikolaos Nikoloudakis, Marianna Hagidimitriou, Andreas Katsiotis

**Affiliations:** 1 Department of Agricultural Science, Biotechnology and Food Science, Cyprus University of Technology, Limassol, Cyprus; 2 Department of Biotechnology, Agricultural University of Athens, Athens, Greece; Aristotle University of Thessaloniki, GREECE

## Abstract

Even though Cyprus was an important crossing point for the westward spread of olive, and one of the primary regions of domestication, its genetic recourses remain uncharted at a great extent. Throughout the centuries, a number of ancient olive trees remain in the same orchards, contributing to Cypriot oleiculture and society. In an attempt to explore this monumental genetic pool, a survey was conducted to identify centennial olive trees in rural provinces of Cyprus. Microsatellites were employed in order to study their genetic composition (including rootstocks when feasible) and to establish possible associations among genotypes. High numbers of specific alleles, suggestive of the distinctiveness of this germplasm, were detected, and both grafting and rootstock propagation was verified. Moreover, it was determined by Bayesian structural and network reticulate analysis that centennial olives can be divided in two discrete genetic clusters having intermediate admixed accessions. Furthermore, it was determined that all contemporary Cypriot cultivars, that were included in the present study, were highly affiliated exclusively to one genetic group, a strong evidence of selection among elite clones. The information acquired from the current study reveals the genetic rareness of this material and its contribution to the current olive germplasm.

## Introduction

The olive tree (*Olea europaea* L.) is the most symbolic species in the Mediterranean Basin due to its ecological, economical and cultural importance [1]. Its contribution to agriculture is recognised since antiquity and no other plant has enjoyed more acknowledgment. Even though the utilization of (mainly) feral olive trees has been reported across the Mediterranean Basin since the Neolithic era [2], it is widely accepted that the domestication of the olive tree began in the Levant approximately 6000 years ago [3] and spread westwards through commercial shipping and land migration across the Mediterranean Basin [4,5]. Nowadays, the olive germplasm constitutes a composite of weedy types (*O*. *europaea* var. *sylvestris*), wild forms (*Olea oleaster* Hoff. Link) and domesticated forms classified as *O*. *europaea* var. *europaea* [2]. Because olive trees can propagate vegetatively, and given the outcrossing nature of *O*. *europaea*, the genotypes spread by cutting or seed migration caused great confusion on cultivar identity and nomenclature [6]. Furthermore, it is likely that olive clones hybridized with oleasters in refugia that endured the glacial Quaternary period [7,8], whereas parallel domestication events [9] have possibly complicated genetic relationships among olives.

In this context, the study of centennial olives can be beneficial for interpreting such a complex history; a history which is the outcome, not only of climatic and biological circumstances, but recently at a great extent anthropogenically driven. Since olive trees have an extensive longevity, monumental trees can be used as milestones of germplasm selection. The immense importance of such plant genetic resources has been only recently appreciated, and an extensive survey on centennial olives has been initiated across the Mediterranean Basin; specifically, in Italy [10], Spain [11], Israel/Palestine [12] and Lebanon [13]. Cyprus is a fairly secluded island located at the eastern Mediterranean Basin, and has been a dispersal passageway of the olive tree [5]. From antiquity (Early Bronze Age) till present, Cypriot oleiculture has thrived and declined during different eras [14], nonetheless, a number of ancient olive trees have withstood through time and hardships. These ‘living fossils’ are now being preserved and maintained all-over Cyprus, and enjoy a significant historical, ornamental and mythological value; hence are classified as ‘monumental’. Unfortunately, new hazards have risen regarding their survival. Human interventions and particularly the ongoing replacement of traditional olive-groves into intensive orchards with introduced cultivars, threatens the continuity of the local ancient olive germplasm. Consequently, the characterization and preservation of the primeval Cypriot olive gene pool should be considered of immense importance. Modern varieties native to Cyprus have been only recently recognised [15] since there hasn’t been a distinction among the cultivated genotypes and all of them—a complex of different genotypes constituting a multiclonal variety—were erroneously referred as 'Ladolia' (i.e. ‘oil producing olive’). In addition, poor information exists regarding the molecular characterization of the modern Cypriot cultivars, thus obstructing the full exploitation capacity and the potential financial impact into the high quality local production [10].

The goal of the present study is to uniquely fingerprint and characterise the Cypriot centennial olive germplasm using microsatellite markers. Furthermore, it is intended to portray the structural patterns of the centennial olive germplasm and to detect the possible admixture among genotypes. Moreover, we focus in the allocation of genotypes studied into defined gene pools, by using reticulate analysis and a model-based Bayesian clustering method. Finally, the possible linkage among the ancient and the current Cypriot germplasm is discussed.

## Materials and methods

### Plant material

**Fig 1:**
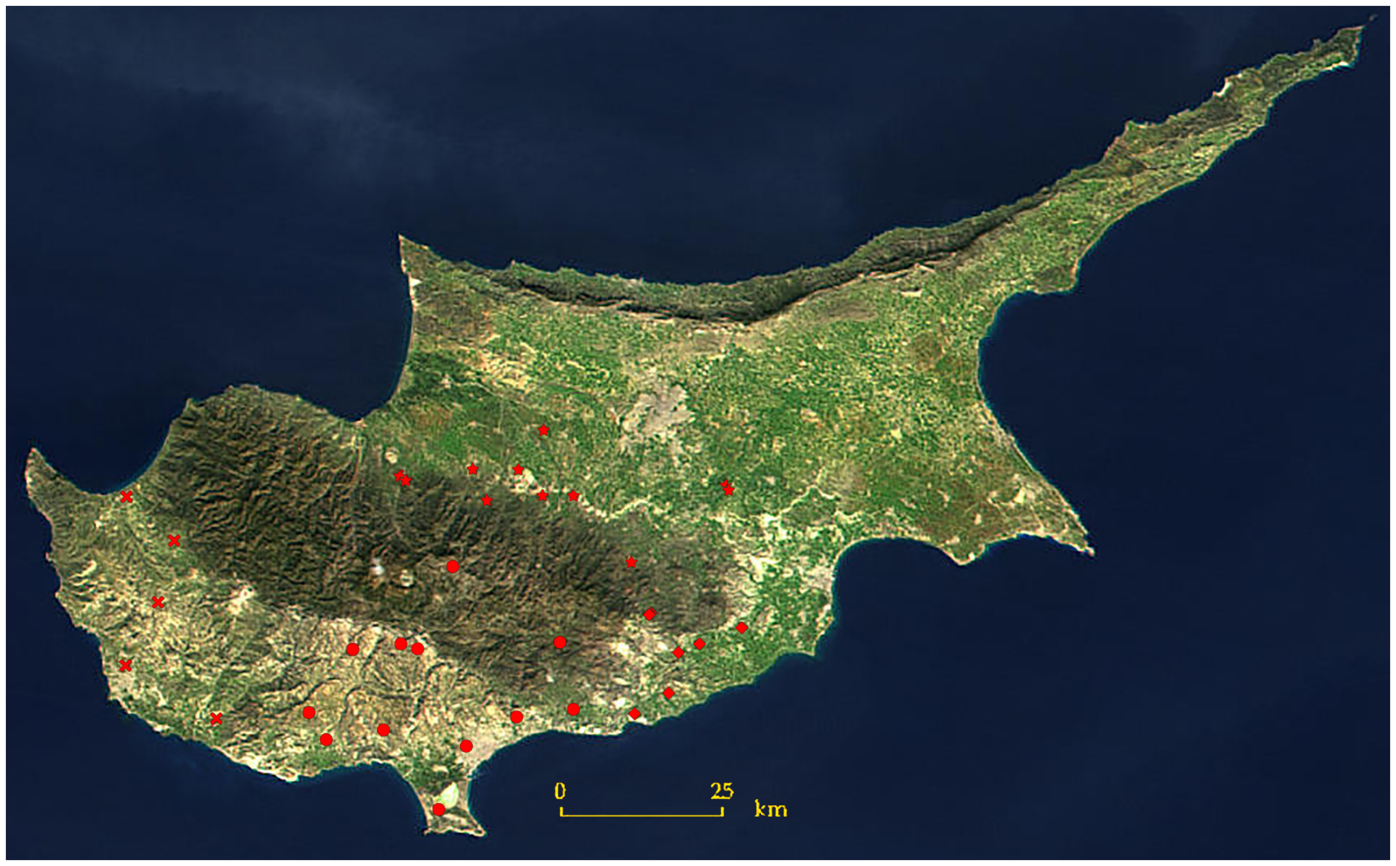
Collection sites of the ancient Cypriot genotypes. Cross, Pafos Prefecture; Circle, Limassol Prefecture; Star, Nicosia Prefecture and Diamond, Larnaka Prefecture. Satellite image acquired from NASA Earth Observatory (public domain): http://earthobservatory.nasa.gov/.

**Fig 2:**
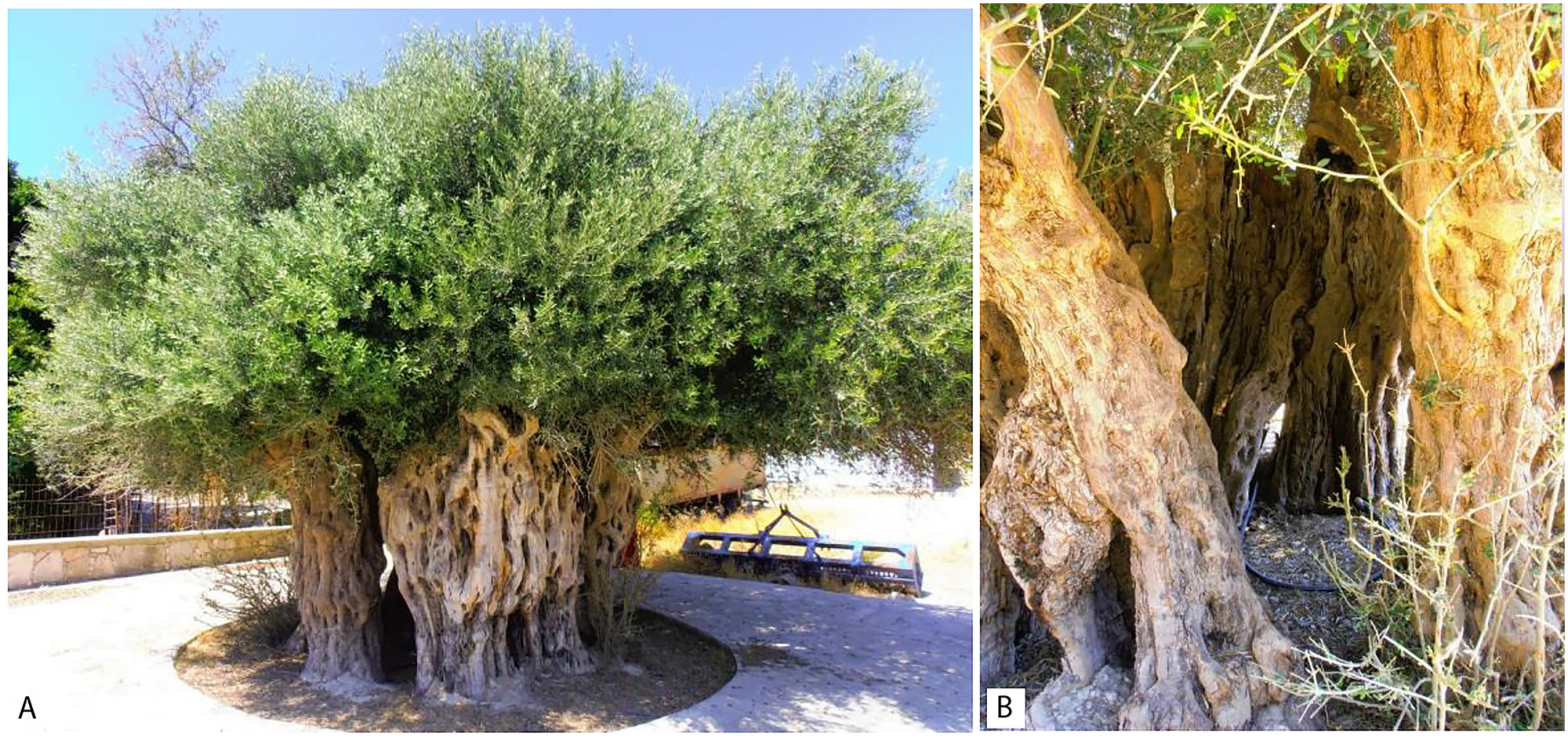
Centennial olive tree sampled in this study. Details of canopy (A) and root suckers (B).

Records from the Cypriot Ministry of Agriculture, Rural Development and Environment were combined with field inquiries in order to discover centennial olive trees. As a result, many of the trees were found from published catalogues of the Cypriot Department of Forests (and are protected as monumental trees), while the remaining were located after interviews with the owners and locals. The countryside was divided in four prefectures (Limassol, Larnaka, Nicosia and Pafos) and after a systematic examination, 52 centennial olive trees were recognized and sampled (Table 1; Fig 1). In several cases, in order to discriminate among grafted and self-rooted trees, leaves from both the canopy and the root suckers (when detected) were sampled (Fig 2). In addition, the monumental olive tree of Ano Vouves (Chania, Greece), which has been estimated to be more than 2000 years old [16], was also included in the analysis. The primary criterion for the selection of the trees was the size of the trunk perimeter that varied from 4.30 to 13 m.

**Table 1:** List of olive trees analyzed. Centennial olives had a trunk perimeter that varied from 4.30 to 13 m. GPS coordinates, Prefecture and Country of sampling were recorded.

No	Accessions	Latitude	Longitude	Origin
1.	'Agios Sozomenos'	35.0525	33.4393	Nicosia
2.	'Aglisides'	34.8541	33.4655	Larnaka
3.	'Akaki'	35.1289	33.1300	Nicosia
4.	'Akrotiri'	34.6021	32.9538	Limassol
5.	'Akti Kyverniti A'	34.7348	33.2851	Larnaka
6.	'Akti Kyverniti B'	34.7372	33.2859	Larnaka
7.	'Anogyra A'	34.7356	32.7330	Limassol
8.	'Anogyra B'	34.7356	32.7333	Limassol
9.	'Avdimou'	34.6990	32.7624	Limassol
10.	'Empa'	34.8018	32.4237	Pafos
11.	'Eptagonia'	34.8346	33.1581	Limassol
12.	'Filousa'	34.9748	32.5054	Pafos
13.	'Flasou B'	35.0651	32.8860	Nicosia
14.	'Flasou C'	35.0654	32.8875	Nicosia
15.	'Flasou D'	35.0594	32.8966	Nicosia
16.	'Germasogia'	34.7300	33.0852	Limassol
17.	'Kato Moni A'	35.0737	33.0865	Nicosia
18.	'Kato Moni B'	35.0740	33.0876	Nicosia
19.	'Klirou A'	35.0378	33.1817	Nicosia
20.	'Klirou B'	35.0377	33.1818	Nicosia
21.	'Klirou C'	35.0384	33.1807	Nicosia
22.	'Kofinou'	34.8315	33.3947	Larnaka
23.	'Kyperounta'	34.9394	32.9773	Limassol
24.	'Lania'	34.8250	32.9177	Limassol
25.	'Lythrododas'	34.9454	33.2783	Nicosia
26.	'Mitsero'	35.0384	33.1292	Nicosia
27.	'Nikokleia'	34.7284	32.5767	Pafos
28.	'Pano Lefkara A'	34.8724	33.3085	Larnaka
29.	'Pano Lefkara B'	34.8743	33.3116	Larnaka
30.	'Polemidia'	34.6900	33.0002	Limassol
31.	'Poli Chrysohous'	35.0354	32.4254	Pafos
32.	'Potamia'	35.0463	33.4435	Nicosia
33.	'Potamiou'	34.8239	32.8077	Limassol
34.	'Psematismenos'	34.7626	33.3418	Larnaka
35.	'Pyrgos'	34.7405	33.1818	Limassol
36.	'Skarinou'	34.8197	33.3586	Larnaka
37.	'Sotira A'	34.7123	32.8606	Limassol
38.	'Sotira B'	34.7120	32.8606	Limassol
39.	'Stroubi'	34.8897	32.4782	Pafos
40.	'Sylikou A'	34.8324	32.8883	Limassol
41.	'Sylikou B'	34.8319	32.8875	Limassol
42.	'Vyzakia'	35.0755	33.0108	Nicosia
43.	'Xiliatos A'	35.0317	33.0350	Nicosia
44.	'Xiliatos B'	35.0319	33.0346	Nicosia
45.	'Xiliatos C'	35.0315	33.0350	Nicosia
46.	'Poli Chrysohous R'	35.0354	32.4254	Pafos
47.	'Aglisides R'	34.8541	33.4655	Larnaka
48.	'Polemidia R'	34.6900	33.0002	Limassol
49.	'Sotira B R'	34.7120	32.8606	Limassol
50.	'Eptagonia R'	34.8346	33.1581	Limassol
51.	'Flasou R'	35.0648	32.8914	Nicosia
52.	cv. 'Analiontas 1'	-	-	Cyprus
53.	cv. 'Analiontas 2'	-	-	Cyprus
54.	cv. 'Evrihou 1'	-	-	Cyprus
55.	cv. 'Evrihou 3'	-	-	Cyprus
56.	cv. 'Flasou'	-	-	Cyprus
57.	cv. 'Kato Drys'	-	-	Cyprus
58.	cv. 'Kiti'	-	-	Cyprus
59.	cv. 'Klirou 1'	-	-	Cyprus
60.	cv. 'Klirou 2'	-	-	Cyprus
61.	cv. 'Korakou'	-	-	Cyprus
62.	cv. 'Lefkara 1'	-	-	Cyprus
63.	cv. 'Paliometocho'	-	-	Cyprus
64.	cv. 'Adramitini'	-	-	Greece
65.	cv. 'Amfisis'	-	-	Greece
66.	cv. 'Chalkidikis'	-	-	Greece
67.	cv. 'Kalamon'	-	-	Greece
68.	cv. 'Koroneiki'	-	-	Greece
69.	cv. 'Lianolia'	-	-	Greece
70.	cv. 'Manaki'	-	-	Greece
71.	cv. 'Megaron'	-	-	Greece
72.	'Elia Vouvon'	35.4871	23.7868	Greece

In order to detect potential genetic relationships among the contemporary and ancient Cypriot olive germplasm, certified varieties by the Cypriot Ministry of Agriculture, Rural Development and Environment were included in the study. In total, 12 Cypriot cultivars from the Gregoriou [15] collection held at the Acheleia (Pafos, Cyprus) experimental station were sampled. Passport data for these cultivars are presented in S1 Table. Finally, nine Greek cultivars, were included in the analysis in order to detect possible similarities among the two germplasms.

### DNA extraction and molecular characterization

Total DNA was extracted from olive leaves using the NucleoSpin Plant II Kit (Macherey-Nagel Düren, Germany). DNA concentration was determined spectrometrically and its quality was established by agarose gel electrophoresis. After an initial screening, 14 carboxyfluorescein (FAM) labeled SSR primer pairs were chosen because of their consistency in amplification and polymorphism (Table 2). Amplification reactions were set up in a 25 μl volume of a mixture containing 20 ng of genomic DNA, 0.75 U *Taq* (Promega, Madison WI, USA), 1X reaction buffer, 320 μM dNTPS and 0,4 μM of each primer. Unambiguous loci where also evaluated when needed with the Type-it Microsatellite PCR Kit (Qiagen, Venlo, Netherlands). PCR amplification was performed using a Bio-Rad PTC-200 thermocycler (Applied Biosystems, Foster City, CA, USA) with the following temperature profile: initial denaturation step for 5 min at 95°C followed by 34 cycles of 45 s at 95°C, 45 s at 50–55°C, 45 s at 72°C and a final elongation step for 10 min at 72°C. SSR primers were selected based on their efficacy and robustness previously described [17–19]. SSR products were separated using an ABI 3130 Genetic Analyzer. Size standard GeneScan 500 LIZ™ (Applied Biosystems, Foster City, CA, USA) was included in each sample to define allele sizes. Data were analyzed using the GeneMapper ^®^ (Applied Biosystems, Foster City, CA, USA) software. Seven olive accessions (10% of the total data set) were analyzed in replicates (DNA extraction, PCR amplification and fragment analysis) to test the reproducibility of allele identification, and constantly included in each run in order to detect possible allele size shifts.

**Table 2:** Genetic variability of olive genotypes and discrimination efficiency of SSR markers used. Observed heterozygosity (*Ho*); expected heterozygosity (*He*); polymorphic information content (*PIC*); probability of null alleles (*F Null*).

*Locus*	*Ho*	*He*	*PIC*	*F(Null)*
GAPU 45	0.917	0.568	0.467	-0.255
GAPU 47	0.958	0.688	0.631	-0.199
GAPU 59	0.917	0.773	0.730	-0.092
GAPU 71a	0.833	0.636	0.559	-0.172
GAPU 71b	1.000	0.600	0.512	-0.272
GAPU 89	0.986	0.675	0.615	-0.220
GAPU 101	0.958	0.618	0.539	-0.244
GAPU 103A	1.000	0.630	0.556	-0.255
UDO99-011	0.000	0.080	0.077	0.683
UDO99-028	0.847	0.580	0.487	-0.206
UDO99-031	0.153	0.256	0.248	0.328
UDO99-039	0.028	0.133	0.129	0.584
UDO99-043	0.972	0.752	0.713	-0.153
UDO99-044	0.875	0.773	0.728	-0.065

### Data analysis

Allele sizes were converted into a data matrix table and a Maximum Likelihood analysis was conducted using the SH-aLRT algorithm and the W-IQ-TREE tool [20]. The level of informativeness was estimated by computing the observed heterozygosity (*Ho*), the expected heterozygosity (*He*), the polymorphic information content (*PIC*) and the probability of null alleles (*F Null*) using the Cervus software [21]. Primers with a probability of null alleles were excluded from the analyses. AMOVA was conducted in order to estimate the degree of genetic differentiation among populations using the GenAlEx 6 software [22]. The significance of the resulting variance components and inter-population genetic distances were tested using 999 random permutations. Reticulate analysis was constructed using the Median-Joining model, as implemented in the SplitsTree4 software [23], under default settings. The Bayesian model-based clustering approach was performed using Structure 2.3.4 [24] in order to identify the genetic structure of the centennial olive germplasm. The structure algorithm was run using the admixture model, with 10 independent replicate runs per K value (number of clusters) ranging from 1 to 10. Each run involved a burning period of 500,000 iterations and a post burning simulation length of 500,000. Validation of the most likely K number and visualization of the aligned clusters was performed using the CLUMPAK application [25].

## Results

### Microsatellite polymorphism and allelic frequency

A total of 52 centennial olive trees along with 20 cultivars were analyzed using 11 out of 14 SSR loci. The SSR profiles led to the identification of 43 different genotypes among the 72 accessions of the present study. Furthermore, the microsatellite profiles from the canopy and from the root suckers differed in four out of six olive trees, which is an indisputable indication of grafting. Replicate PCR amplifications and fragment analyses revealed the reproducibility of the detected SSR loci, including rare alleles (data not shown). The analysis revealed a total of 109 alleles, ranging from two to 14 alleles for the UDO99-011 and UDO99-043/GAPU 103A loci, respectively, and an average of 7.79 alleles per locus. The GAPU 103A locus showed the highest number of specific alleles (seven). Allelic frequencies ranged from 0.007 (specific alleles) to 0.96, presenting polymorphism for each locus. In addition, 27 specific alleles were detected among the centennial olives (Table 3); four were observed in the centennial olive scions, five in the rootstocks, two in the Cypriot cultivars and 16 in Greek germplasm.

**Table 3:** Sizes and frequency of alleles detected in the olive germplasm.

SSR locus	Number of alleles	Allele size (bp) detected per genotype*													
GAPU 45	4	181 (8)	183 (66)	196 (68)	198 (2)										
GAPU 47	9	161 (3)	183 (2)	185 (55)	187 (8)	189 (6)	191 (58)	193 (6)	**195** **(1)**	203 (5)					
GAPU 59	8	208 (36)	210 (28)	212 (46)	214 (24)	**216** **(1)**	218 (6)	220 (2)	**256** **(1)**						
GAPU 71a	4	208 (5)	210 (55)	212 (19)	214 (65)										
GAPU 71b	6	119 (4)	122 (65)	124 (64)	**126** **(1)**	128 (4)	142 (6)								
GAPU 89	10	161 1(0)	163 (3)	171 (63)	175 (2)	177 (2)	**179** **(1)**	195 (4)	203 (2)	205 (5)	207 (52)				
GAPU 101	11	189 (2)	191 (67)	193 (5)	197 (2)	199 (59)	201 (2)	**205** **(1)**	207 (3)	**213** **(1)**	**215** **(1)**	**217** **(1)**			
GAPU 103A	14	**133** **(1)**	**135** **(1)**	**139** **(1)**	150 (66)	**159** **(1)**	161 (3)	171 (2)	175 (58)	177 (4)	179 (2)	185 (2)	**197** **(1)**	**209** **(1)**	**211** **(1)**
UDO99-011	2	136 (6)	140 (138)												
UDO99-028	8	124 (6)	**130** **(1)**	**132** **(1)**	**143** **(1)**	**148** **(1)**	150 (62)	152 (70)	154 (2)						
UDO99-031	8	**104** **(1)**	108 (7)	110 (124)	112 (4)	**121** **(1)**	140 (3)	154 (3)	**158** **(1)**						
UDO99-039	4	171 (2)	178 (134)	180 (4)	191 (4)										
UDO99-043	14	**172** **(1)**	174 (3)	176 (3)	178 (7)	202 (2)	204 (3)	**206** **(1)**	**208** **(1)**	210 (3)	212 (55)	214 (4)	216 (43)	218 (16)	220 (2)
UDO99-044	7	**123** **(1)**	125 (4)	140 (2)	144 (34)	146 (34)	148 (42)	150 (27)							
**Sum**	**109**														

*: the number of different olive genotypes having the allele is indicated under the alleles sizes. Private alleles are highlighted in bold.

Table 2 summarizes the genetic variability for the olive genotypes. In order to estimate the discriminating capability of the SSR markers, the polymorphic information content (*PIC*) was calculated. The highest *PIC* value was 0.730, while the lowest was 0.077 for loci GAPU 59 and UDO99-011, respectively, with an average of 0.499. The likelihood of null alleles incidence ranged from -0.272 to 0.683. For the 11 out of the 14 loci analyzed, the observed heterozygosity was significantly higher than the expected values (Hardy-Weinberg equilibrium). Finally, a considerable heterozygosity deficiency was detected for UDO99-011, UDO99-031 and UDO99-039 loci. These results suggest a selection of elite clones and a declination from random mating (panmixia).

Analysis of molecular variance revealed that a high proportion (92%) of the total genetic diversity was allocated within the populations. The highest variability was recorded for the ancient Cypriot genotypes (SS = 288,344), followed by modern Cypriot and Greek cultivars (SS = 76,167 and SS = 70,667, respectively). Fst values also suggested the presence of significant divergence between populations (Fst = 0.113, p = 0.001).

### Clustering analysis

**Fig 3:**
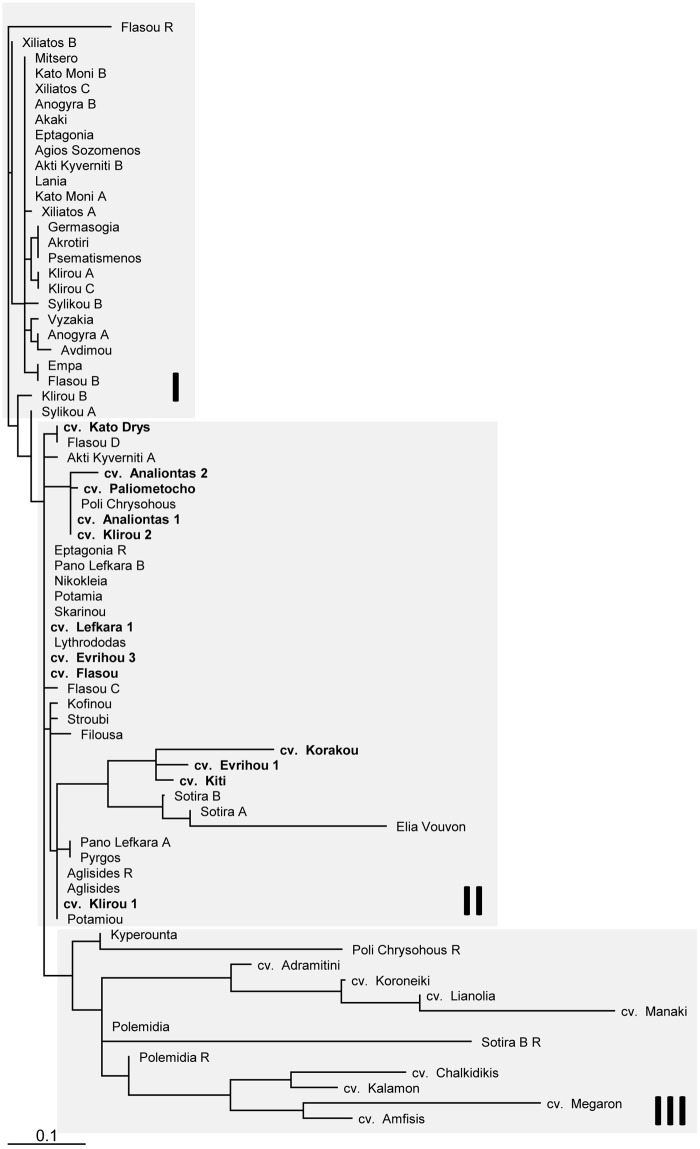
Maximum likelihood dendrogram depicting genetic similarities among the 72 genotypes studied. Three discrete groups are distinguished with separated shaded boxes. Contemporary Cypriot cultivars are in bold.

The majority of the Cypriot olive genotypes (58 out of 63) were organized in two distinctive clusters (I, II), while all Greek taxa and a few Cypriot olive trees were organized in a third (III) diverse group (Fig 3). Thirty-seven out of 72 genotypes shared uniform allelic patterns and were organized in nine separate clusters, each having two to 10 entries. Cluster I contained 26 ancient olive trees, but no modern cultivars, while Cluster II contained 33 taxa; 21 centennial olive trees and all 12 contemporary Cypriot cultivars. In general, olive trees that were sampled in the same village were found to be similar (probably clones of the same cultivar); in some cases, with noteworthy declinations. Hence, olive trees from the villages of Kato Moni, Xiliatos, Klirou and Anogyra were grouped within the A cluster and the genotypes from the municipality of Pano Lefkara were present in the B cluster. In contrast, samples from the villages of Flasou, Sylikou and Akti Kyverniti were mainly dispersed among the major clusters, while the accessions from the Sotira region were related to the Greek ancient olive 'Elia Vouvon' (Fig 3).

Four out of six centennial olive trees that were tested were found to be grafted ('Poli Chrysochous', 'Sotira B', 'Eptagonia' and 'Polemidia'), indicating that the majority of the olive trees of the present study are grafted. Still, several modern cultivars shared the same genetic composition with centennial olives, revealing an active participation of the ancient Cypriot olive germplasm to the current via vegetative/clonal propagation. For instance, cv 'Klirou 2' and cv Analiontas 1' were identical to the accession of 'Poli Chrysochous'; furthermore, cv 'Klirou 1' was indistinguishable from the 'Aglisides' and 'Potamiou' genotypes, while cv 'Lefkara 1', cv 'Flasou' and cv 'Evrihou 3' were integrated in the second biggest genetic group containing eight entries (Fig 3). Finally, low genetic affinity to the core of the olive germplasm was observed for the rootstocks 'Sotira B R', 'Poli Chrysochous R', 'Flasou R', the scions 'Sotira A' and 'Sotira B' and for the cvs 'Evrihou 1', 'Kiti' and 'Korakou'.

### Coalescent modeling

**Fig 4:**
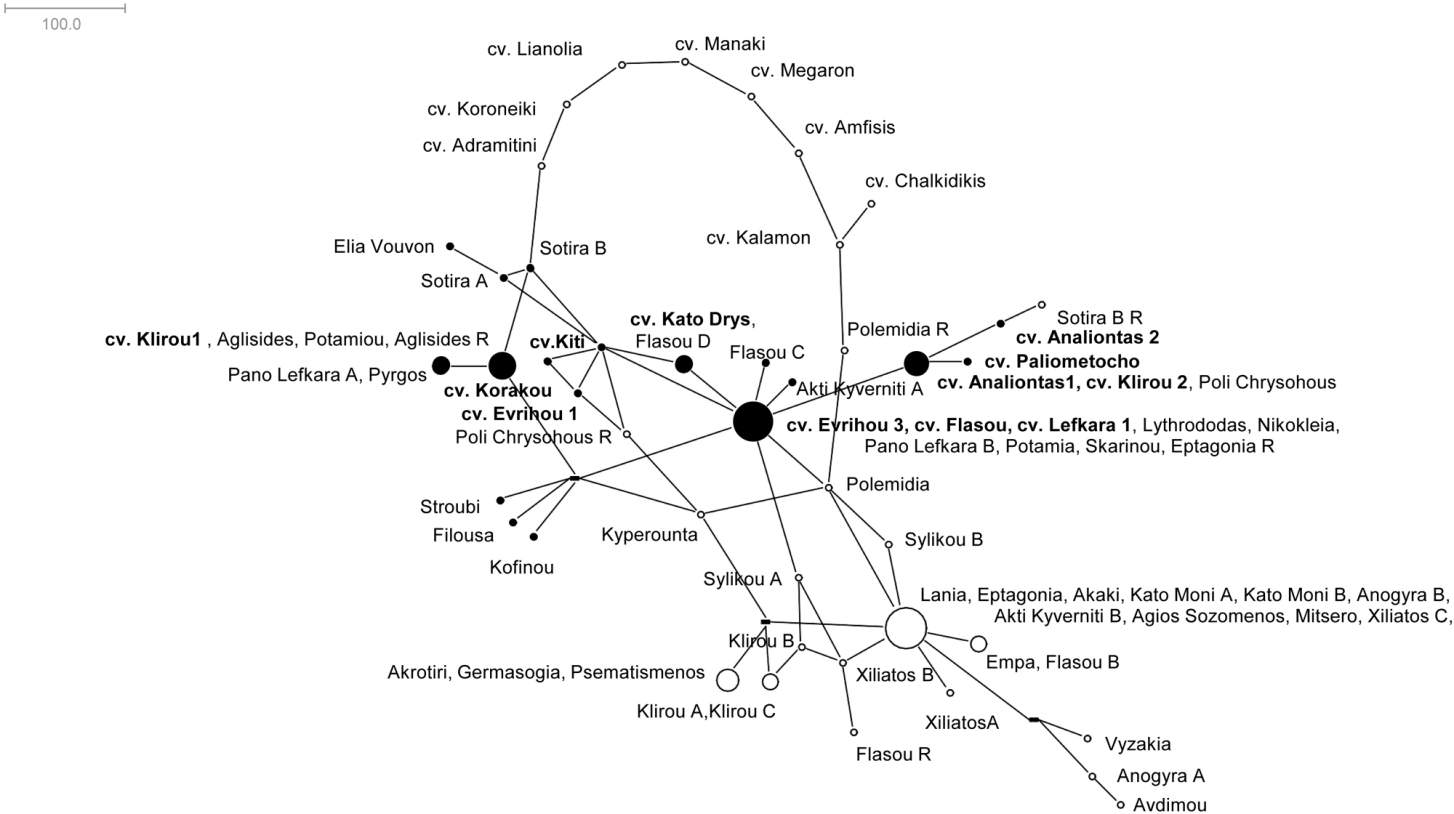
Reticulate analysis based on microsatellite data. Colouring of open nodes (black) matches the accession’s assignment to the second cluster (according to the dendrogram). Contemporary Cypriot cultivars are in bold.

In order to additionally resolve the genetic relationships among the olive entries, a reticulate analysis was employed and a network was constructed by coalescent simulations (Fig 4). Based on the frequency and the topology of the combined microsatellite haplotypes, all entries were clustered into a complex network. Reticulate and linear relations were depicted equally within the olive germplasm. The Greek cultivars were positioned as outgroups having 'Sotira A' and 'Polemidia R' accessions as an inter-node to the Cypriot olive germplasm. Furthermore, no distinction was possible between the cultivars 'Flasou' 'Lefkara 1' and 'Evrihou 3', as well as, among the cultivars 'Analiontas 1' and 'Klirou 2'. In general, the association of the genotypes was in agreement to the Maximum Likelihood Analysis. Entries had primarily direct connections between them, eventhough a number of them participated in complex reticulate links. Remarkably, only a portion of the centennial entries have contributed to the current genetic material, thus serving as the only material for the selection of all contemporary Cypriot olive germplasm. The two largest nodes, that contained nine and ten entries, seemed to participate actively to the construction of diverge genetic germplasm via the multiple connections with several different genotypes. Most of the rootstocks were positioned externally to the core of the olive entries, thus underlining their lack of genetic affinity, but without ruling out the possible contribution to the establishment of the current cultivars. For instance, 'Poli Chrysochous R' and 'Sotira B R' were directly linked to contemporary Cypriot cultivars ('Kiti'/'Evrihou 1' and 'Analiontas 2', respectively). As in the case of the dendrogram, no apparent relation among the entries of the first group and the current Cypriot cultivars was possible. Nevertheless, it appears that the current Cypriot cultivars encompass rich genetic variability since they were not grouped but are scattered throughout the network. In addition, genotypes of this group actually had more composite intra- and inter-genetic associations (and lower genetic similarity values).

### Inference of Cypriot olive germplasm structure

**Fig 5:**
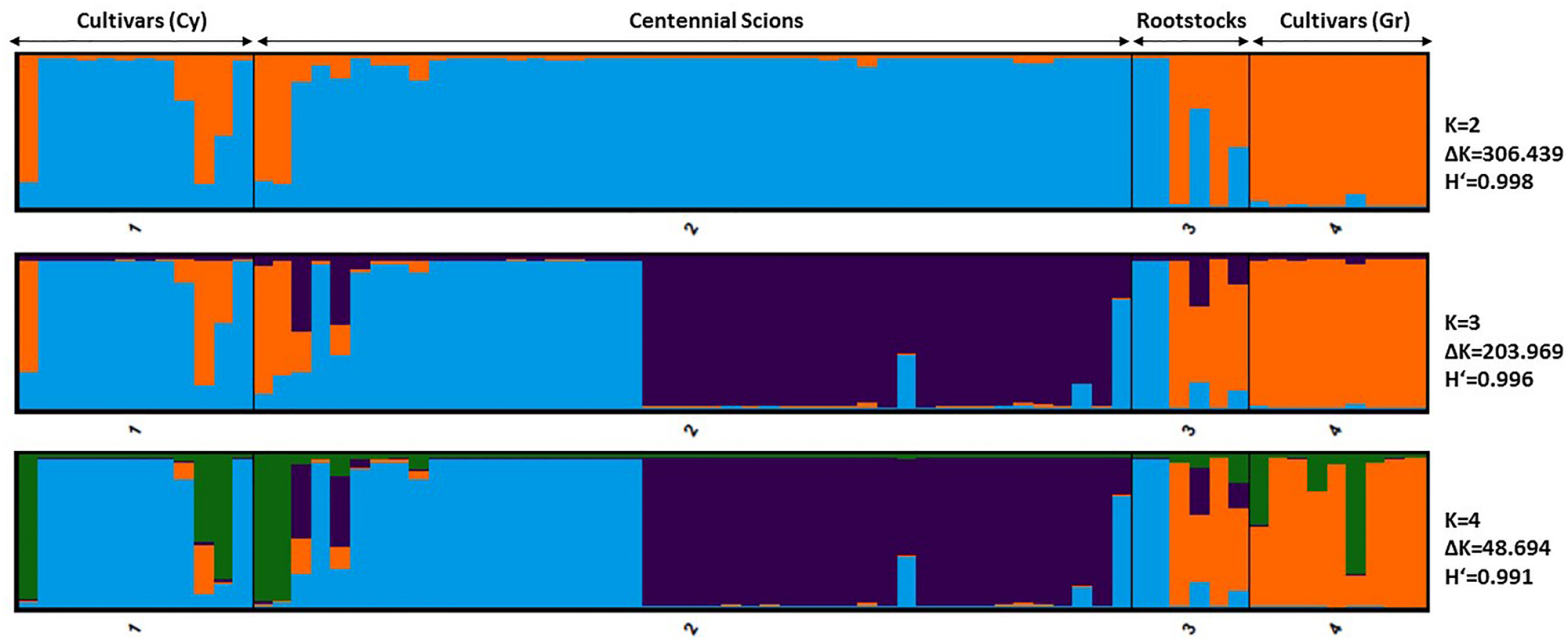
Bayesian cluster analysis of the optimum K clusters. Percentages of participation of each accession studied to genetic clusters (different colour), as inferred at K = 2, K = 3 and K = 4.

The pattern of genetic admixture and germplasm structure was additionally portrayed using a Bayesian-based approach and the visual outline of substructures among the olives is presented in Fig 5. A range from K = 1 to K = 10 was tested and the posterior probability for each value of K was computed using the estimated log likelihood of K. The structure analysis demonstrated a major break in the slope of likelihood values at K = 2 (ΔK = 306.439, Mean similarity coefficient: 0.998), K = 3 (ΔK = 203.969, Mean similarity coefficient: 0.996), and a further minor break at K = 4 (ΔK = 48.694, Mean similarity coefficient: 0.991), signifying that splitting the germplasm into two or three clusters should correspond to the optimal subdivision of data. For K = 2, all ancient olive genotypes were depicted as a homogenous group (blue colour) with the exception of 'Sotira A' and 'Sotira B' genotypes, while most of the rootstocks were affiliated to the Greek cultivars, except 'Aglisides R' and 'Eptagonia R' (orange colour). Furthermore, for K = 3 the Cypriot centennial olives (scions) were divided into two major genetic pools (blue and purple colour). The majority of genotypes were homogenous and had a membership value higher than 0.8, with the exception of 'Xiliatos B', 'Kyperounta', 'Polemidia', 'Klirou B' and 'Sylikou A' that had admixed genotypes. Almost all contemporary cultivars were clustered in Group I (blue colour) except cvs 'Evrihou 1', 'Kiti' and 'Korakou' that had little genetic resemblance to the rest. All Greek cultivars were homogenous and were allocated to Group II (orange colour) having genetic affinity to a restricted number of current Cypriot cultivars and to some Cypriot rootstocks. The up-scaling of population size (K = 4), did not severely distorted the genetic relations among the accessions, even though it provided a more detailed outline. Nevertheless, two ancient Cypriot olive trees 'Sotira A' and 'Sotira B' emerged as a separate cluster and were genetically identical to the modern Cypriot cvs 'Evrihou 1', 'Kiti' and 'Korakou', that were affiliated to the rootstock germplasm and the Greek cultivars (for K = 3). At higher K values, more complex groups were produced making classification criteria complicated.

## Discussion

### Monumental olive trees individuality

Out of 43 different genotypes identified, only a few centennial trees had an identical genetic profile to contemporary cultivars, while the rest were different (Fig 3). This is in accordance to Baldoni et al. [26], Erre et al. [27], Diez et al. [11], and Chalak et al. [13] that have reported a low embodiment percentage (less than 10%) of the ancient olive germplasm to contemporary local cultivars. The large number of specific alleles identified and the high heterogeneity reported in the current study were similar to other sets of cultivated and wild olives [28,29]. This richness in genetic resources is probably the outcome of the early domestication of olive trees in Cyprus that occurred in antiquity, while genetic variability accumulated through time [30]. Hence, these entries represent an unexploited genetic pool [11].

Identical genotypes were revealed mainly from entries that were collected in close geographical proximity (same village), with the exception of the Kato Moni village. On the contrary, Hosseini-Mazinani et al. [31] identified clones within and between the regions sampled. Therefore, in some instances, accessions that were collected from the same territory (Xiliatos, Anogyra, Klirou etc) were grouped together. However, there were occurrences where olive trees from the same region (Flasou, Sylikou and Akti Kyverniti) clustered in different groups. This is in accordance to Gregoriou [15] and Banilas [30] who reported that taxa from the same village had little morphological resemblance and low genetic similarity. The large number of identical genotypes detected among the centennial olive trees underlines that these clusters represent primeval clonally propagated varieties which were intensively exploited for oleiculture [31]. The network analysis permitted the simultaneous depiction of genetic relations among the monumental olive trees and thus, it can be concluded that several genotypes may have a determined role to the establishment of the contemporary olive germplasm. Therefore, the core of the two genetic groups could have acted as the precursor, either via hybridization with local and/or foreign material [8,32–34], or accumulation of point mutations [11,35] contributing to the proliferation of genetic variability in Cypriot olive trees [31]. The 'Sotira A' and 'Sotira B' accessions were found diverged in all analyses conducted, and were placed as an internode among the Greek and Cypriot olive germplasm, having genetic affinity to the listed cultivars 'Evrihou 1' 'Kiti' and 'Korakou'. This is a clear indication that there were more than one cultivar types in the primary olive orchards. Barazani et al. [12] performed an extensive survey among ancient olive trees of the Levant (scions and rootstocks) and determined that the majority of the trees were grouped in the same multi-locus lineage; it is likely that additional cultivars might have been ‘hidden’. Based on the reticulate and the structure analysis, a few accessions were depicted as intermediates among the two major clusters ('Klirou B', 'Sylikou A' and 'Xiliatos B'). These genotypes could be the result of hybridization of different populations. Such an admixture is also reported by Hosseini-Mazinani et al. [31].

Grafting has been described as the transition to domestication and this technique was developed almost four millennia ago [36]. The SSR profiles among the canopy and root-suckers samples, differed significantly in four out of six centennial olive trees; a strong indication of tree crafting. This observation is in accordance to Diez et al. [11] and Barazani et al. [12] who detected that the majority of ancient trees in Spain and in Israel/Palestine respectively, were grafted; however, this was not the case in olive trees in Lebanon [13]. The 'Aglisides' entry was not found to be grafted although the possibility of sampling above the point of grafting due to the trunk architecture cannot be discarded [12]. Allelic differences were detected between rootstocks and scions for entries 'Eptagonia'/'Eptagonia R' and 'Polemidia'/'Polemidia R', eventough the most divergent genotypes were detected between 'Sotira B'/'Sotira B R' and 'Poli Chrysohous'/'Poli Chrysohous R'. Furthermore, genetic affinity among the rootstocks and the Greek genotypes was detected, an indication that not only scions, but also rootstocks were selected in historical times [12].

The high genetic variability of rootstocks raises questions about their origin. For an explanation, several possibilities can be proposed: primarily, as suggested by Diez et al. [11] for the Iberian Peninsula, rootstock variation could be attributed to wild populations of olive (since *O*. *europaea* var. *sylvestris* was common in the Levant [3]. Alternatively, the bimodal frequency distribution of genetic distances in ancient rootstocks of the Levant, points towards that rootstock variation is the result of sexual reproduction [12]. Consequently, it is plausible that scions were grafted on trees which either sprouted from seeds of cultivated trees, or emerged spontaneously as feral trees in the olive orchards. Admixed genotypes have probably played a role in the establishment, at least in the case of Cypriot olive germplasm. Finally, there is also the possibility that mutation could have contributed to the enrichment of the genetic diversity of rootstocks [12]

### Two major gene pools

The majority of the centennial olive trees and the local multiclonal varieties of 'Ladolia' were clustered in two discrete groups in all the analyses carried out. The grouping highlights the close intra population relations, possible due to common ancestry and the diversification among clusters [30]. Structure analysis revealed that the majority of Cypriot olive trees are homogenous with minor exceptions. Admixed genotypes could be the result of seedlings or of sexually propagated material originating from diverged populations. Haouane et al. [37] used microsatellites to analyze the genetic structure of olives in the Mediterranean Basin and determined three distinct genetic pools; (a) the western Mediterranean, (b) the central Mediterranean and (c) the eastern Mediterranean. Cypriot cultivars were rather homogenous for the low level population complexity (all genotypes were affiliated to the eastern Mediterranean gene pool), but as the genetic structure became more composite, an admixture was revealed and a close relationship also to Greek cultivars was manifested.

This similarity could either highlight their common genetic background since Cyprus is referred as a possible passage for the dispersal of olives westward of the Levant [26,29], or it could be a reintroduced genetic material from the central Mediterranean and possibly used as rootstocks [12]. Since, oleiculture in Cyprus counts for millennia, both theories could be valid. In a more recent study by Chalak et al. [13], a distinction within the genotypes of Levant was evident. Syrian olive varieties diverged from all others, while most of the accessions from Cyprus and Lebanon (apart from individuals from the Bekaa region) were clustered into one gene pool. The centennial Lebanese trees of Bcheale were astonishingly affiliated to the Cypriot olive germplasm. Consequently, this pattern strongly indicates the ancient exchange of olive germplasm among Cyprus and Lebanon, given the long history of earlier trading transversely the Mediterranean [1,5]. However, it cannot be uncritically ruled out, that the discrimination of olive accessions in two distinctive groups may be the result of the ongoing selection for olive usage (table/oil producing olives), as already demonstrated [11,32,38–41].

### The establishment of the contemporary Cypriot cultivars ('Ladolia')

Extensive genetic differences were detected among the Cypriot cultivars. Morphological descriptors [15], as well as, molecular markers [30] have validated the divergence existing within the contemporary olive germplasm. This can be attributed both to accumulated somatic mutations or sexual reproduction. Hybridization of local and feral olives to indigenous [8,29,32] or foreign genetic material [13] has been shown to develop new cultivars. Based on the morphological and genetic diversity of the 'Ladolia' complex, it has been proposed that it is constituted by a mixture of non bred cultivars and must be multi-clonal [15,30]. This is also the case for the local Lebanese cultivar 'Baladi' which is also composed by different genotypes. Nevertheless minor genetic differences have also been detected. Somatic mutations have been reported for olive in the recent past using RAPD, AFLPs and SSRs [30,35,42,43]. The possibility of mutations is considerable for clonally propagated centennial olives due to the longevity of the trees, reflecting the significant accumulation of genetic diversity [11–13,35,44]. Moreover, there is also the possibility of genetic mosaicism due to the accumulation of mutations [45]. Repetitive nucleotide regions are very variable and can accumulate mutations without phenotypic alterations in morphological and agricultural characteristics [46]. Unfortunately, the correlation of single mutations and phenotypic variation remains unanswered and especially the cutoff value that constitutes a different variety [47]. Further studies are needed in order to define the factors that affect evolution of clonally propagated species, such as olive [48,49] and future studies focusing in centennial olives could assist to that direction. In the study of Haouane et al. [35], Cypriot cultivars were clustered with genotypes of eastern Mediterranean countries such as Syria, Lebanon and Egypt. According to Chalak et al. [13], the majority of Cypriot cultivars held at the Marrakech genebank have genetic similarity to cultivar 'Baladi' as well as to other Lebanese centennial olive accessions, while a small group of Cypriot accessions has homology to Syrian entries.

Since there is little resemblance of contemporary Cypriot cultivars to the second cluster of ancient genotypes, it can be concluded that the former germplasm has suffered a reduction of genetic variability. It is known that the domestication process involves a selective bottleneck and a shift in the direction of fixing alleles associated to desirable agronomic characters [50]. Hence, the current Cypriot accessions are not representative of the available existing Cypriot olive genetic pool and as a consequence these accessions reserved in genebanks [13,31,35], could in fact, be a small subset.

## Conclusions

The study of Cypriot centennial olive trees was fruitful since the collection of this unique and diverse material can elucidate the agricultural practises and the domestication procedures that led to the establishment of the current olive germplasm. The extensive genetic diversity observed, points out that these genotypes are probably not clones of the ancient Cypriot cultivar 'Ladolia', but have a diverse origin. Cypriot scions were divided in two discrete clusters and modern cultivars were directly affiliated to one, while rootstocks were mainly affiliated to Greek genotypes. Based on the data of the current study it is safe to formulate the conclusion that oleiculture in Cyprus has a complex history. It is possible that hybridization between local or/and foreign material, followed by selection of elite genotypes has repeatedly occurred in different eras with the goal of ameliorating the existing genotypes. Rootstocks of centennial olives retain their ‘hidden’ rich genetic diversity and further focused studies can provide clues for the spread of olive germplasm around the Mediterranean basin. Hence, the collection and maintenance of centennial olives is a critical first step towards breeding and evolutionary unravelling of the oleiculture history through different eras.

## Supporting information

S1 Table Passport data for modern Cypriot cultivars. (XLSX)
